# Supplementary figures and images for: Evaluation of Radiolabeled Girentuximab In Vitro and In Vivo
Source: Pharmaceuticals (Basel). 2018 Nov 28;11(4):132. doi: 10.3390/ph11040132 (PMC6316122; doi:10.3390/ph11040132)

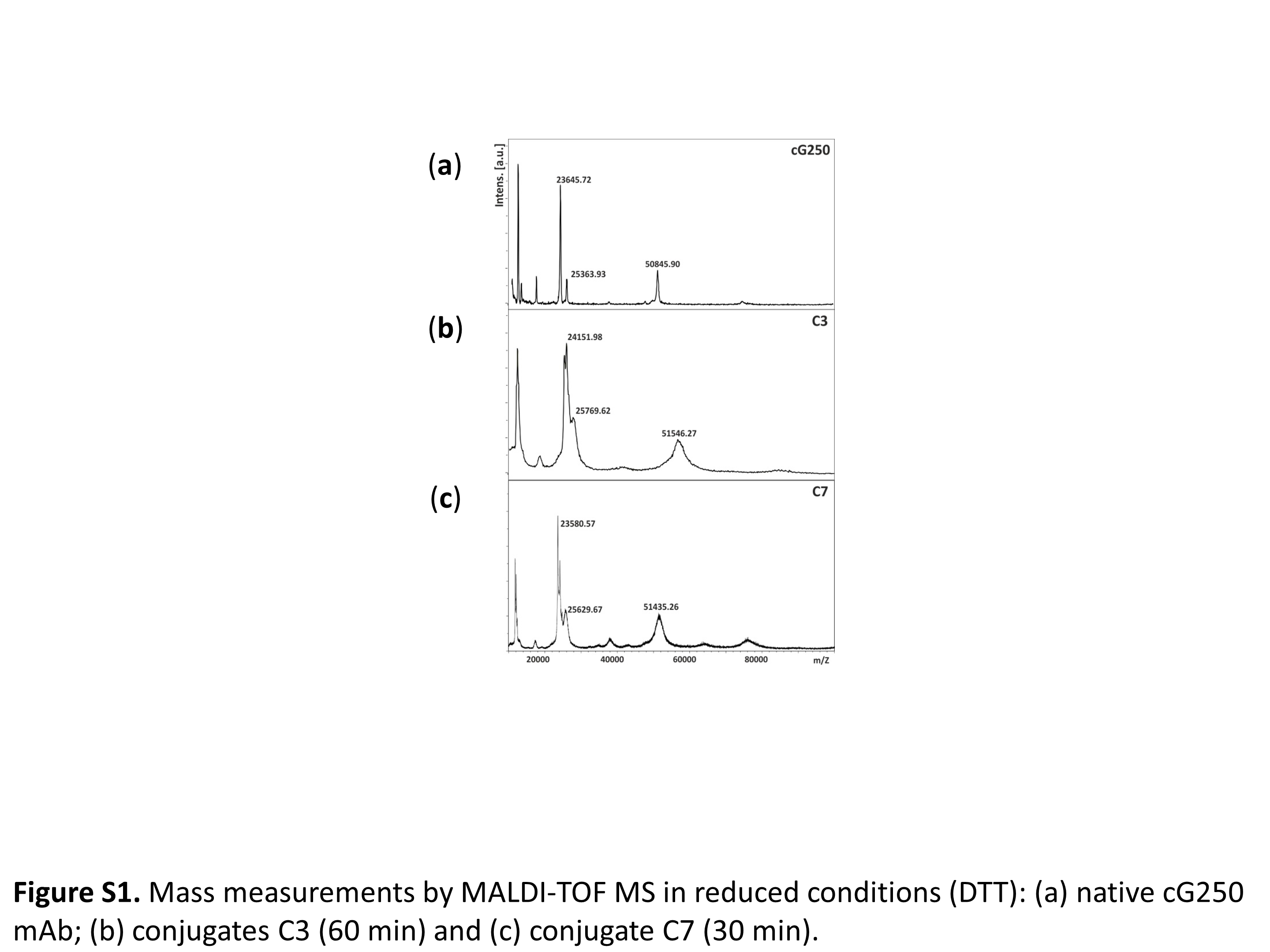

Supplement: Supplementary file 1 [file pharmaceuticals-11-00132-s001.zip › pharmaceuticals-387209-proofreading-suppl/Figure S1 .TIF]

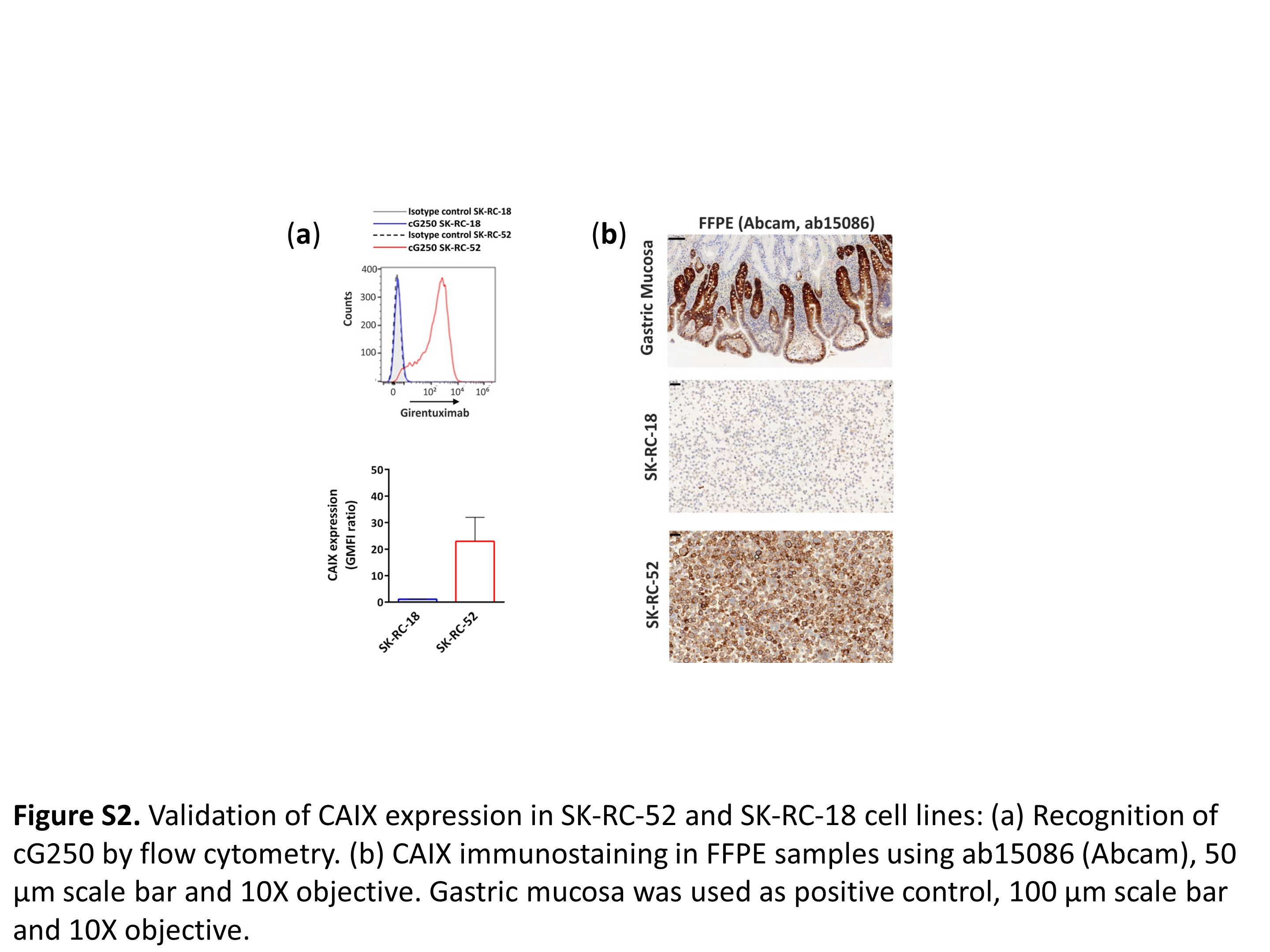

Supplement: Supplementary file 1 [file pharmaceuticals-11-00132-s001.zip › pharmaceuticals-387209-proofreading-suppl/Figure S2.TIF]

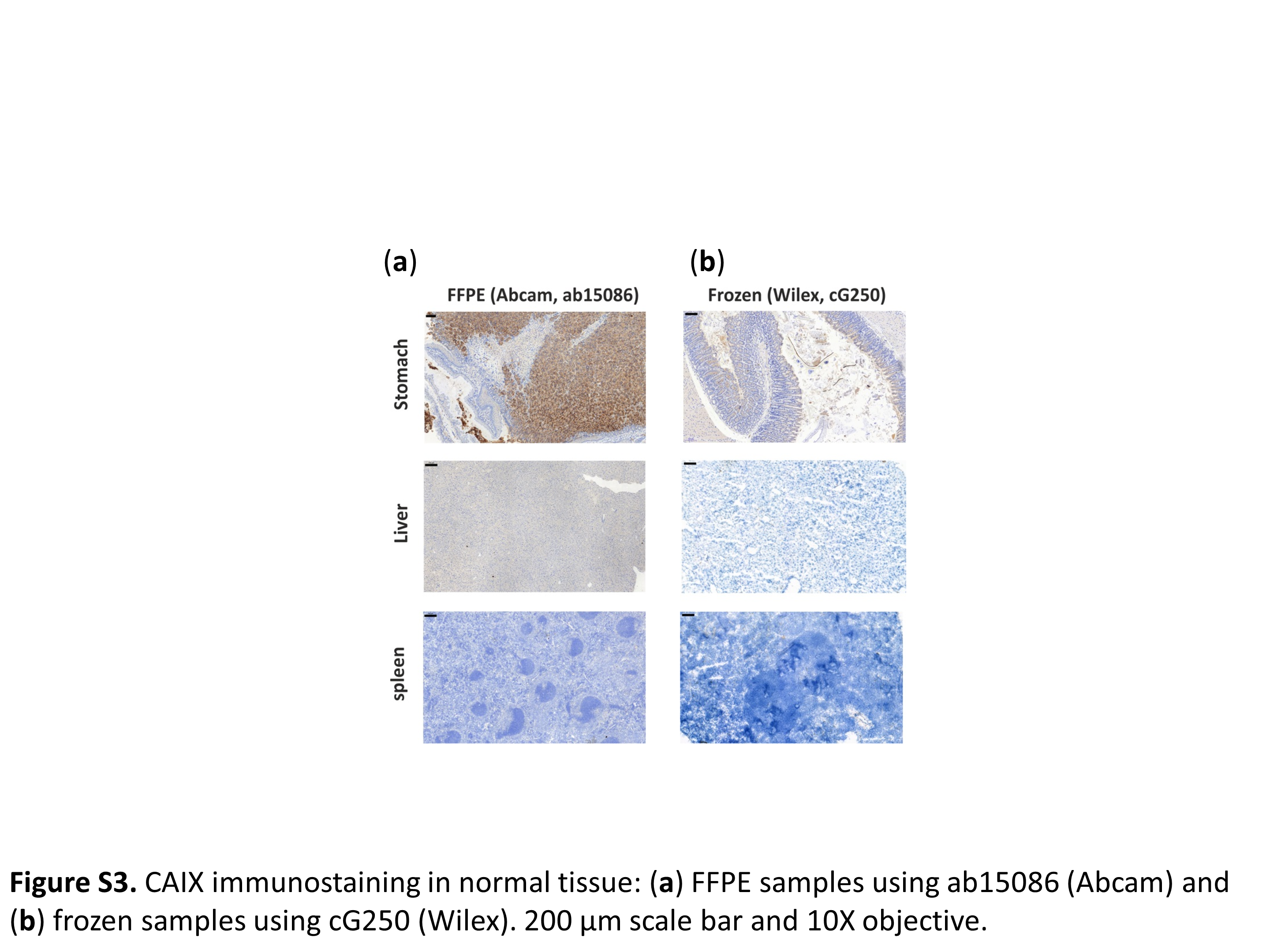

Supplement: Supplementary file 1 [file pharmaceuticals-11-00132-s001.zip › pharmaceuticals-387209-proofreading-suppl/Figure S3.TIF]

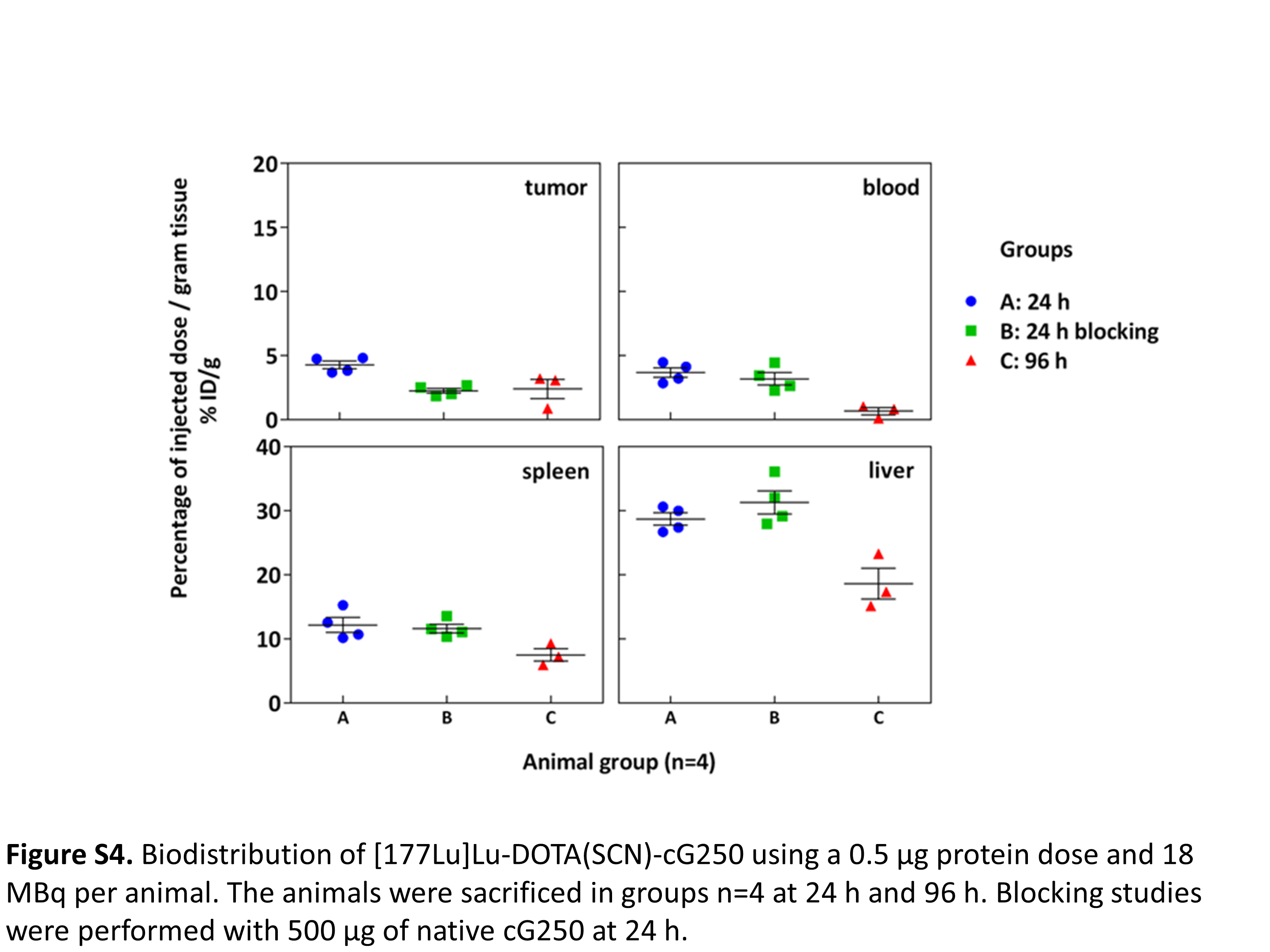

Supplement: Supplementary file 1 [file pharmaceuticals-11-00132-s001.zip › pharmaceuticals-387209-proofreading-suppl/Figure S4.TIF]

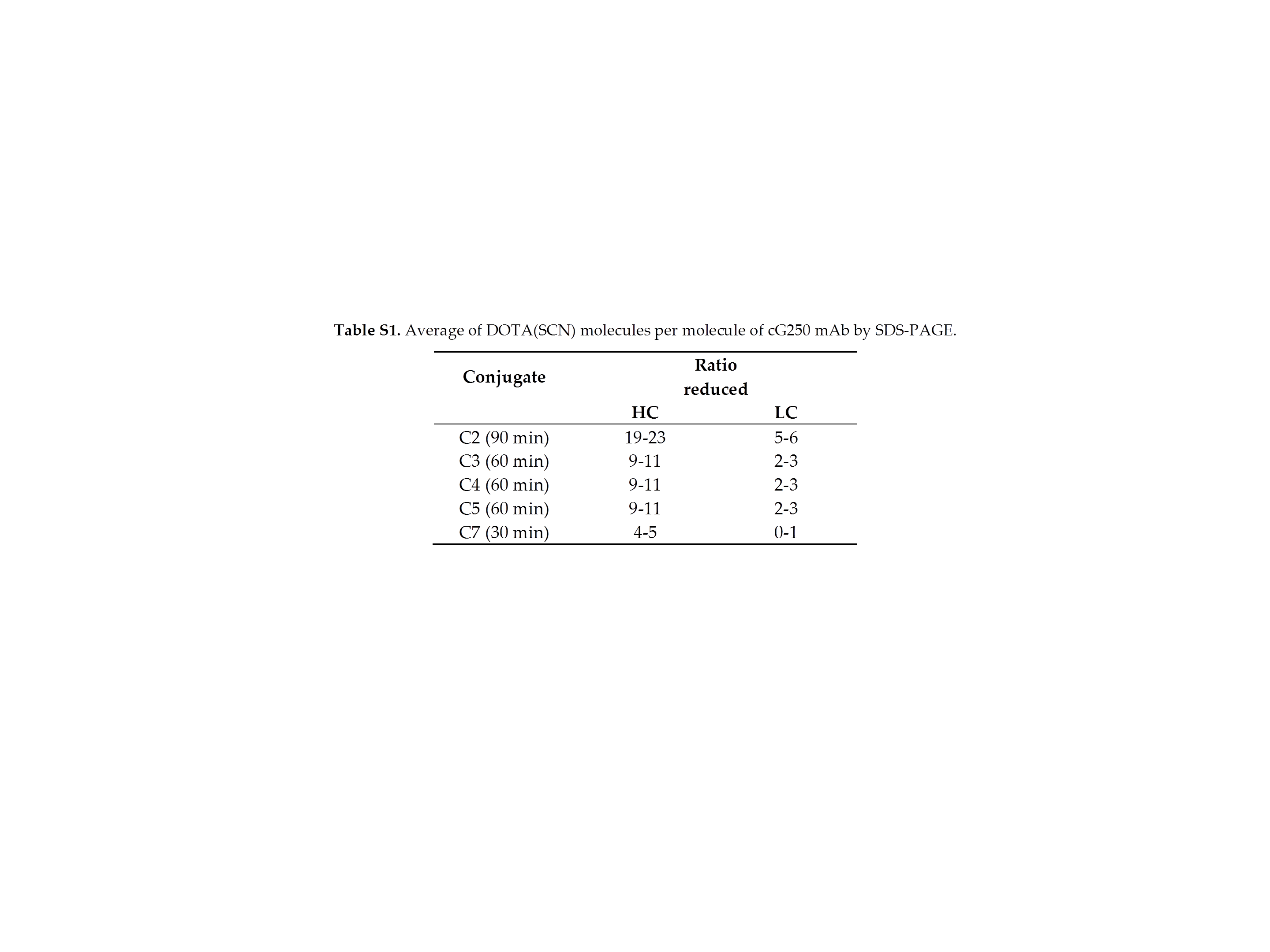

Supplement: Supplementary file 1 [file pharmaceuticals-11-00132-s001.zip › pharmaceuticals-387209-proofreading-suppl/Figure S5.TIF]
